# Return to work after cancer treatment of gynecologic cancer in Japan

**DOI:** 10.1186/s12885-016-2627-0

**Published:** 2016-07-29

**Authors:** Keiichiro Nakamura, Hisashi Masuyama, Takeshi Nishida, Junko Haraga, Naoyuki Ida, Masayuki Saijo, Tomoko Haruma, Tomoyuki Kusumoto, Noriko Seki, Yuji Hiramatsu

**Affiliations:** Department of Obstetrics and Gynecology, Okayama University Graduate School of Medicine, Dentistry and Pharmaceutical Sciences, 2-5-1 Shikata-cho, Kita-ku, Okayama, 700-8558 Japan

**Keywords:** Return to work, Job change, Gynecologic cancer survivors

## Abstract

**Background:**

Gynecologic cancer is one of the most common malignant diseases in working-age women. This study investigated whether several characteristics influence return to work after treatment of gynecologic cancer.

**Methods:**

We investigated the correlations between return to work and several other characteristics in 199 gynecologic cancer survivors. Questionnaires were distributed to patients with cancer (≥1 year after treatment and age of <65 years) who visited Okayama University. Logistic regression analysis and receiver operating characteristic curves were used to determine whether each characteristic influenced return to work (no return to work or job change) in these gynecologic cancer survivors.

**Results:**

For all patients, the mean age at the time of diagnosis was 47.0 years, and the average number of years after treatment was 4.5. Forty-four patients (53.7 %) who were non-regular employees continued to be employed at the same workplace. Non-regular employment had a significantly higher area under the curve (AUC) (0.726) than other characteristics in terms of negatively affecting return to work. Additionally, non-regular employment tended to have a higher AUC (0.618) than other characteristics in terms of job changes.

**Conclusions:**

Non-regular employment was the variable most likely to negatively affect return to work and job changes in employed patients who underwent treatment for gynecologic cancer.

## Background

The incidence of gynecologic malignancy has increased in recent years in Japan, with an estimated 30,964 newly diagnosed patients in 2009 [1]. The Japan Society of Obstetrics and Gynecology reported that about 70 % of all patients diagnosed with cancer in Japan were of working age (20–64 years old) [2]. Gynecologic cancer is one of the most common malignant diseases in working-age women, and improvements in the management of gynecologic cancer have increased the survival rate in patients with this disease. Several studies have reported that an average of 60 to 67 % of patients who had worked before their cancer diagnosis returned to work after the initial treatments [3–7]. However, the patients with cancer for whom return to work was investigated and validated included few patients with gynecologic cancer; thus, the applicability of such an investigation of these patients has not been established. This study aimed to clarify the status of return to work among gynecologic cancer survivors.

## Methods

### Study population

Questionnaires were distributed to gynecologic cancer survivors (≥1 year after treatment and age of <65 years) who visited Okayama University for consultation from 28 May 2015 to 28 December 2015. All patients were informed about the survey by their consultant doctors and provided written informed consent to participate in this study. All answers were voluntary. Completed questionnaires were collected using in-hospital collection boxes. This study protocol was approved by the Institutional Review Board of Okayama University Hospital (No. 1504-001). The dataset comprised responses from 199 gynecologic cancer survivors who were employed and working at the time of their cancer diagnosis. Table 1 shows the distribution of the questionnaires with gynecologic cancer survivors (type of employments, work days per week, work time per day, number of people at workplace, personal income, household income, return to work, the work day per week after treatment, the working hours after treatment, person income after treatment, physical and psychological uneasiness after treatment).

**Table 1:** Questionnaires for gynecologic cancer survivors

Did you have employment at the time of diagnosis?	1. Yes	2. No				
What kind of work at the time of diagnosis did you do?	1. Self-employed	2. Publicly employed	3. Regularly employed	4. Non-regularly employed		
How many days did you work per week at the time of diagnosis?	1. 1 day/week	2. 2 day/week	3. 3 day/week	4. 4 day/week	5. 5 day/week	6. ≥ 6 day/week
How many hours did you work per day at the time of diagnosis?	1. < 3 h/day	2. 3–5 h/day	5. 6–8 h/day	6. > 8 h/day		
How many people did you work in workplace at the time of diagnosis?	1. ≤ 5 peoples	2. 6–10 peoples	3. 11–20 peoples	4. 21–30 peoples	5. 31–50 peoples	6. > 50 peoples
How much did you have income (10,000 yen) at the time of diagnosis?	1. <103	2. 103–149	3. 150–299	4. 300–499	5. ≥ 500	
How much did you have household income (10,000 yen) at the time of diagnosis?	1. <300	2. 300–499	3. 500–699	4. 700–999	5. 1,000–1,499	6. ≥ 1,500
Did you have return to work after treatment?	1. Same reinstatement	2. Job change	3. No return to work			
What kind of employment at the job change after treatment?	1. Self-employed	2. Publicly employed	3. Regularly employed	4. Non-regularly employed		
How many days did you return to work after treatment?	1. <1 months	2. 1–3 months	3. 4–6 months	4. 7–12 months	5. >12 months	
Did you change in the working hours after treatment?	1. Increase	2. Decrease	3. No change			
Did you change person income after treatment?	1. Increase	2. Decrease	3. No change			
What did you feel uneasiness after treatment? (Multiple answers allowed)	1. Physical uneasiness	2. Psychological uneasiness				

### Study variables

The type of employment at diagnosis was categorized according to the patients’ responses as follows: self-employed, publicly employed (teacher, dietician, care person), regularly employed (permanently employed), or non-regularly employed (part-time workers, temporary workers, contract-based workers, and dispatched workers). The patients also provided information on the following characteristics: age, marital status, children or no children, cancer site, cancer stage, treatment duration, employment pattern, work days per week, work time per day, number of people at workplace, personal income, household income, physical uneasiness, psychological uneasiness, and return to work (no return to work or change in job). Finally, we examined the correlations between job changes and reinstatement time and between work time and personal income.

### Statistical analysis

Statistical analyses were performed using the Mann–Whitney *U*-test. A receiver operating characteristic (ROC) curve was generated, and the area under the curve (AUC) was calculated to evaluate the discriminatory ability of each scoring system. We also examined the data in cross-tabulated form to explore return to work (no return to work or change in job). Analyses were performed using SPSS software, version 20.0 (IBM Corp., Armonk, NY USA). A *P* value of <0.05 was considered statistically significant.

## Results

A total of 265 gynecologic cancer survivors responded to the questionnaires at the time of diagnosis. At diagnosis, 199 patients were employment, and 66 patients were non-employment. 199 gynecologic cancer survivors who were employed and working at the time of their cancer diagnosis’ characteristics are summarized in Table 2. The mean age at the time of diagnosis was 47.0 years (median, 46.4 ± 9.8 years; range, 25–64 years), and the average number of years after treatment was 4.5. A total of 144 patients (72.4 %) were married, and 138 patients (69.3 %) had children. The diagnoses were cervical cancer (*n* = 105, 52.8 %), endometrial cancer (*n* = 64, 32.2 %), and ovarian cancer (*n* = 30, 15.0 %). More patients had early-stage cancer (*n* = 170, 85.4 %) than advanced-stage cancer (*n* = 29, 14.6 %). A high percentage of patients had undergone only surgery (*n* = 85, 42.7 %); fewer patients had undergone surgery and chemotherapy (*n* = 50, 25.1 %), surgery and radiation with/without concurrent chemotherapy (*n* = 37, 18.6 %), and radiation therapy with/without concurrent chemotherapy (*n* = 27, 13.6 %). At diagnosis, 82 patients (41.2 %) were non-regularly employed, 68 (34.2 %) were regularly employed, 31 (15.6 %) were self-employed, and 18 (9.0 %) were publicly employed.

**Table 2:** Patient characteristics

Age at diagnosis	Median, 46.4	Range, 25–64
	Numbers	(%)
Marry		
Yes	144	72.4
No	55	27.6
Children		
Yes	138	69.3
No	61	30.7
Cancer site		
Cervical cancer	105	52.8
Endometrial cancer	64	32.2
Ovarian cancer	30	15
Stage		
Early	170	85.4
Advanced	29	14.6
Treatment		
Surgery	85	42.7
Surgery + Chemotherpy	50	25.1
Surgery + Radiation (included CCRT)	37	18.6
Radiation (included CCRT)	27	13.6
Employment pattern		
Self-employed	31	15.6
Publicly employed	18	9
Regularly employed	68	34.2
Non-regularly employed	82	41.2

*CCRT* concurrent chemoradiotherapy

**Fig. 1:**
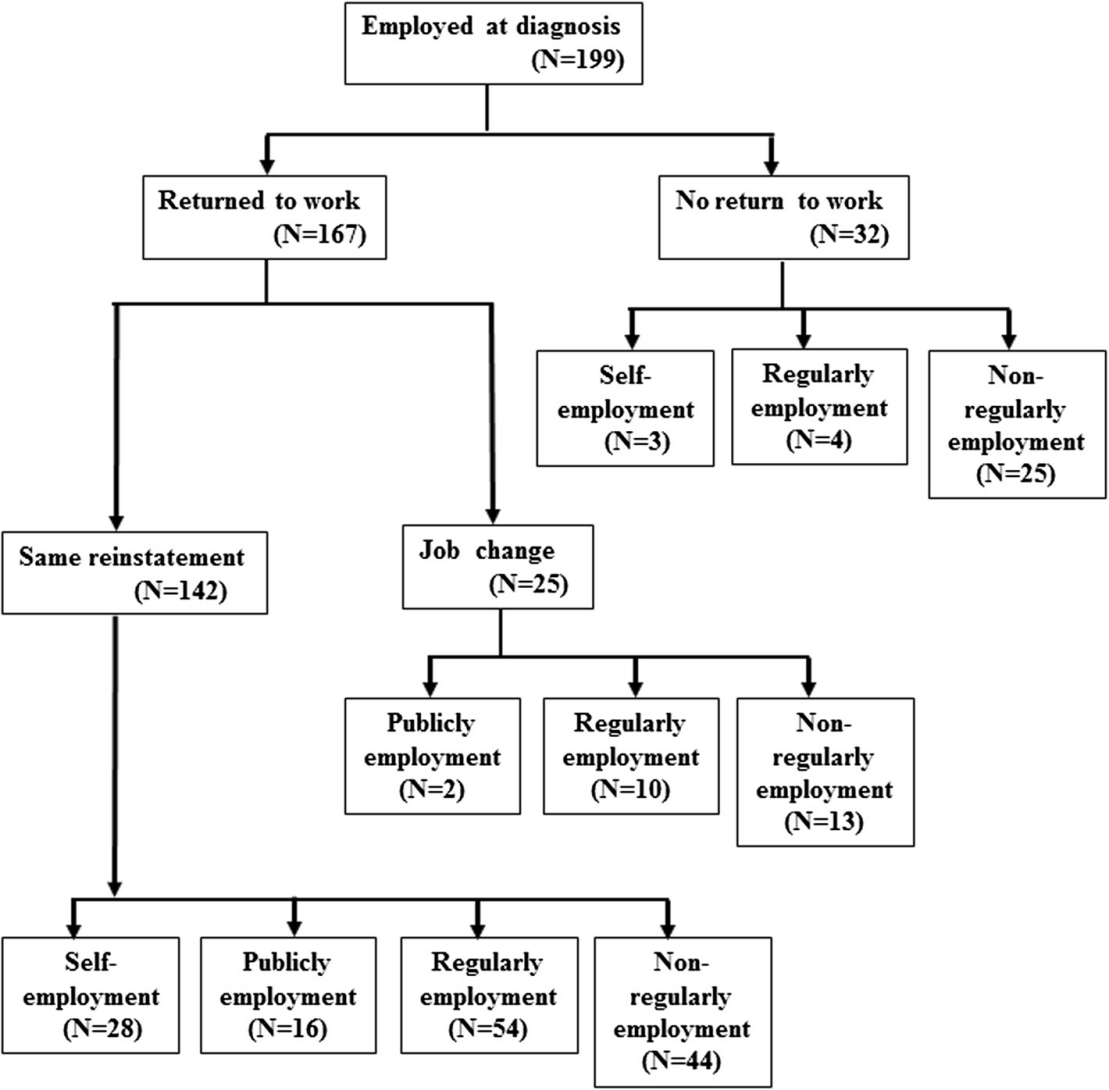
A total of 199 patients with gynecologic cancer were working at the time of cancer diagnosis

In this study, 32 patients (16.1 %) did not return to work and 25 patients (12.6 %) changed their job. These patients were investigated in terms of their relationship with each employment pattern. Patients who did not return to work (including because their business closed) were distributed as follows: self-employed (*n* = 3, 9.7 %), regularly employed (*n* = 4, 5.9 %), and non-regularly employed (*n* = 25, 30.5 %). Patients who changed their jobs were distributed as follows: publicly employed (*n* = 2, 11.1 %), regularly employed (*n* = 10, 14.7 %), and non-regularly employed (*n* = 13, 15.9 %). Interestingly, of the patients who were able to return to the same workplace, 28 (90.3 %) were self-employed, 16 (88.9 %) were publicly employed, 54 (79.4 %) were regularly employed, and 44 (53.7 %) were non-regularly employed (Fig. 1).

**Fig. 2:**
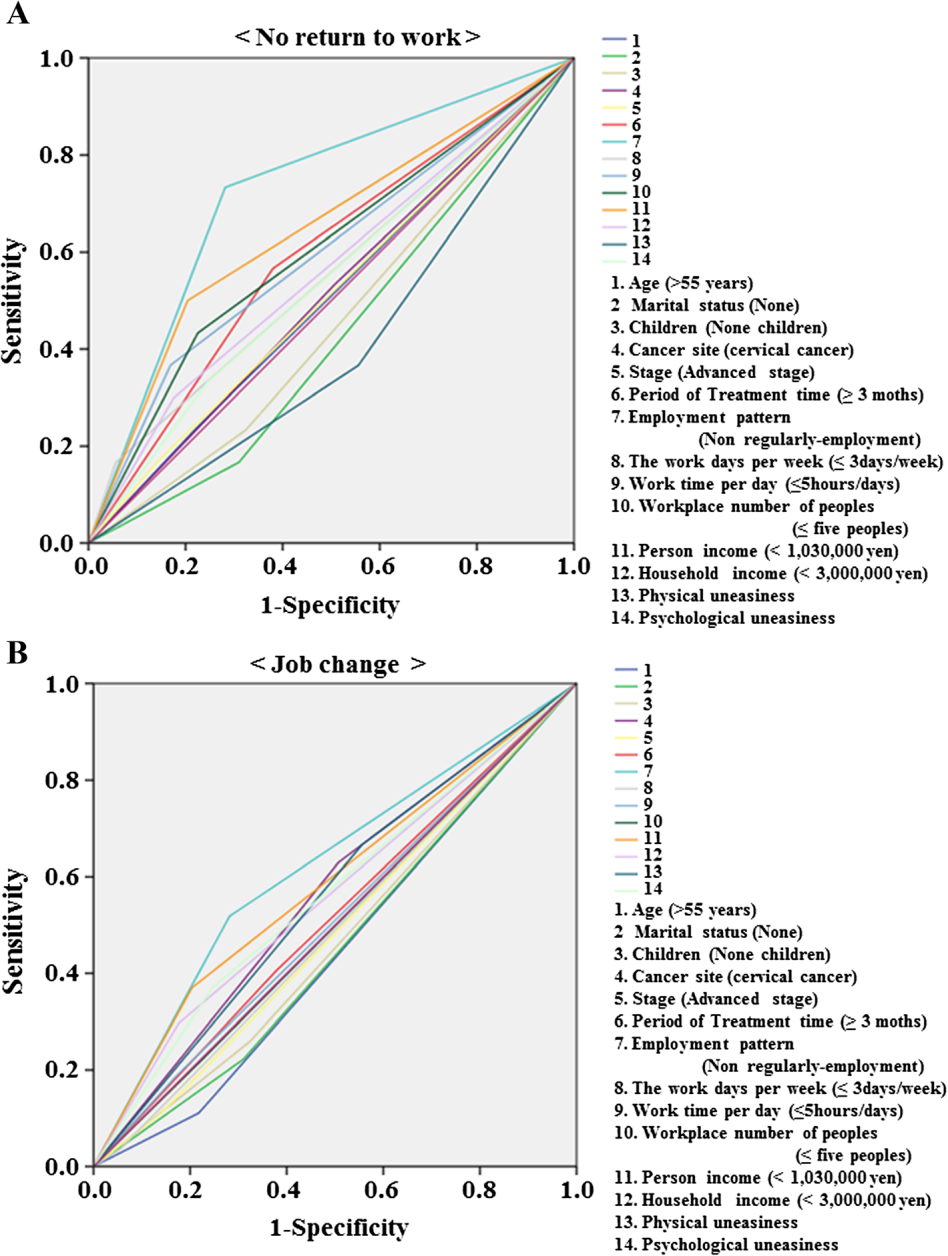
Receiver operating characteristic curve analyses to predict no return to work or job change. **a** No return to work (same reinstatement (*n* = 142) vs no return to work (*n* = 32)). **b** Job change (same reinstatement (*n* = 142) vs job change (*n* = 25)). Correlations among all characteristics: 1. Age, 2. Marital status, 3. Children, 4. Cancer site, 5. Cancer stage, 6. Treatment duration, 7. Employment pattern, 8. Work days per week, 9. Work time per day, 10. Number of people at workplace, 11. Personal income, 12. Household income, 13. Physical uneasiness, and 14. Psychological uneasiness

We investigated correlations among each characteristic; i.e., age, marital status, children, cancer site, cancer stage, treatment duration, employment pattern, work days per week, work time per day, number of people at workplace, personal income, household income, physical uneasiness, psychological uneasiness, and no return to work or job change. We used ROC curve analyses to predict no return to work or job change. Non-regularly employed patients had a significantly higher AUC (0.726) than did other characteristics in terms of negatively affecting return to work. Additionally, non-regular employment tended to have a higher AUC (0.618) than did other characteristics in terms of job change. Moreover, personal income had a significantly high AUC (0.648) in terms of negatively affecting return to work (Fig. 2 and Table 3).

**Table 3:** Comparison of AUC for no return to work and job change

A. Not returned to work (Same reinstatement (*N* = 142) vs Not returned to work (*N* = 32))			
Period	AUC	95%Cl	*P*-value
Age (>55 years)	0.508	0.393–0.622	0.897
Marital status (None)	0.428	0.321–0.536	0.218
Children (None children)	0.455	0.344–0.565	0.436
Cancer site (Cervical cancer)	0.513	0.399–0.627	0.821
Stage (Advanced stage)	0.513	0.398–0.628	0.824
Period of Treatment time (≥3 moths)	0.593	0.480–0.706	0.109
Employment pattern (Non regularly-employment)	0.726	0.625–0.827	<0.001*
The work days per week (≤3 days/week)	0.555	0.436–0.675	0.343
Work time per day (≤5 h/days)	0.599	0.480–0.717	0.089
Workplace number of peoples (≤ Five peoples)	0.604	0.487–0.721	0.074
Person income (<1,030,000 yen)	0.648	0.532–0.763	0.011*
Household income (<3,000,000 yen)	0.562	0.444–0.680	0.287
Physical uneasiness	0.405	0.294–0.516	0.103
Psychological uneasiness	0.543	0.427–0.660	0.455
B. Job change (same reinstatement (*n* = 142) vs job change (*n* = 25))			
Period	AUC	95%Cl	*P*-value
Age (>55 years)	0.446	0.334–0.559	0.378
	0.456	0.341–0.571	0.471
Children (None children)	0.468	0.351–0.584	0.595
Cancer site (Cervical cancer)	0.561	0.444–0.678	0.313
Stage (Advanced stage)	0.485	0.368–0.602	0.807
Period of Treatment time (≥3 moths)	0.514	0.394–0.633	0.823
Employment pattern (Non regularly-employment)	0.618	0.499–0.738	0.051
The work days per week (≤3 days/week)	0.49	0.373–0.608	0.874
Work time per day (≤5 h/days)	0.508	0.388–0.628	0.894
Workplace number of peoples (≤ Five peoples)	0.498	0.379–0.617	0.979
Person income (<1,030,000 yen)	0.583	0.460–0.706	0.172
Household income (<3,000,000 yen)	0.56	0.437–0.683	0.323
Physical uneasiness	0.555	0.439–0.672	0.364
Psychological uneasiness	0.562	0.440–0.684	0.308

*AUC* area under the curve

*p<0.05

**Fig. 3:**
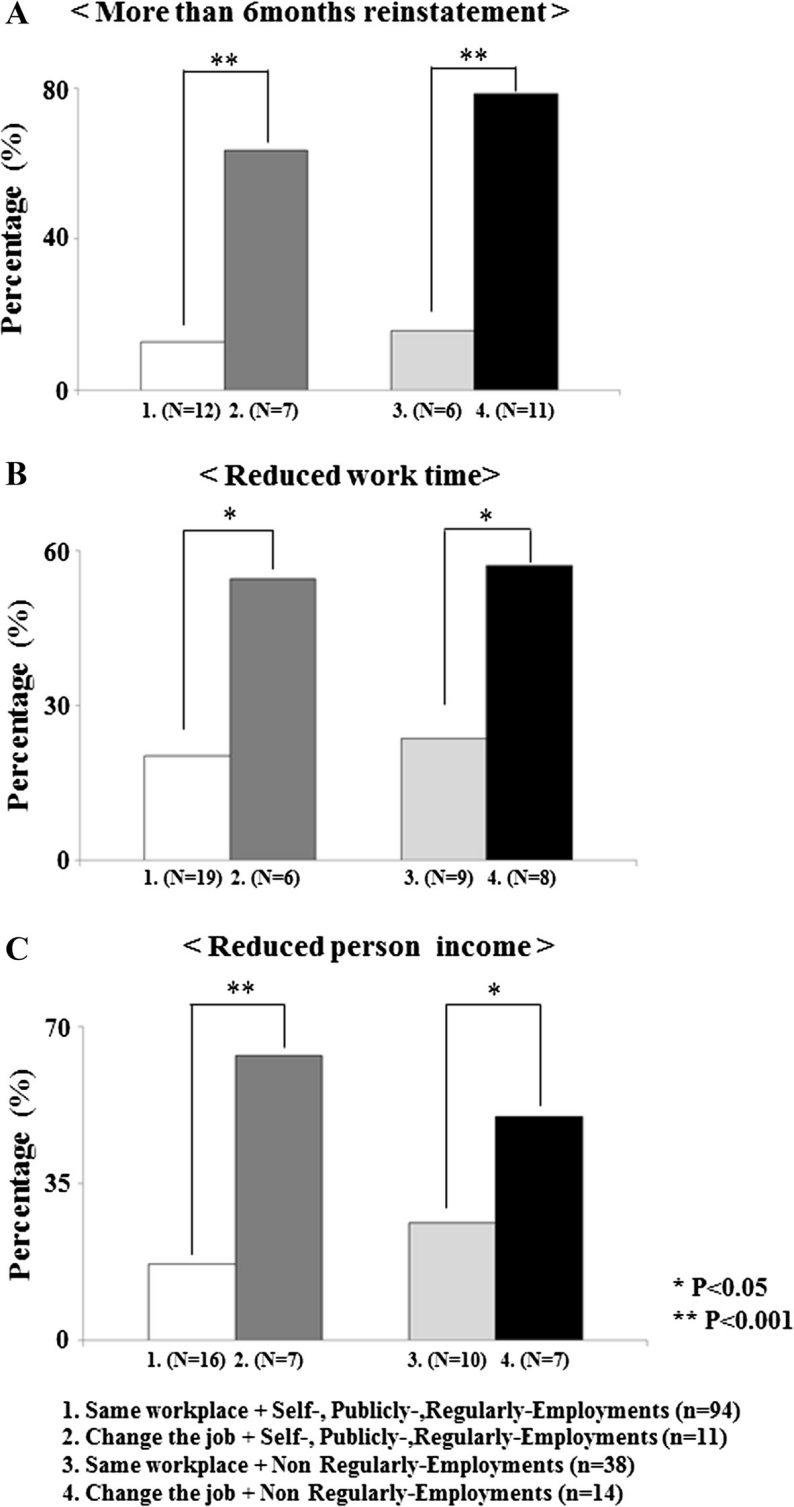
Correlation of employed workplace and employment with post-treatment reinstatement time, work time, and personal income. **a** More than >6 months reinstatement after treatment. **b** Reduced work time after treatment. **c** Reduced personal income after treatment. 1. Same workplace plus self-, public, or regular employment (*n* = 94). 2. Job change plus self-, public, or regular employment (*n* = 11). 3. Same workplace plus non-regular employment (*n* = 38). 4. Job change plus non-regular employment (*n* = 14)

To examine the correlations of employed workplace (same workplace/job change) and employment (non-regular employment/self-, public, and regular employment) with the reinstatement time, work time, and personal income, we divided return to work (including a job change) after treatment into four groups: Group 1, same workplace plus self-, public, or regular employment (*n* = 94); Group 2, job change plus self-, public, or regular employment (*n* = 11); Group 3, same workplace plus non-regular employment (*n* = 38); and Group 4, job change plus non-regular employment (*n* = 14). The percentage of patients with a >6-month reinstatement was significantly higher in Group 2 than Group 1 (*p* < 0.001) and was significantly higher in Group 4 than in Group 3 (*p* < 0.001). The percentage of reduced work time was significantly higher in Group 2 than in Group 1 (*p* < 0.05) and was significantly higher in Group 4 than in Group 3 (*p* < 0.05). There was a significant difference in the percentage of reduced personal income between Groups 2 and 1 (*p* < 0.001) and between Group 4 and Group 3 (*p* < 0.05, respectively) (Fig. 3).

## Discussion

Cancer treatment options are improving, and the number of cancer survivors thus continues to grow. Returning to work after treatment is important for the patient’s quality of life, financial security, restoration of a stable social environment, and the feeling of normality in both patients and their family. Returning to work can improve the quality of life of cancer survivors, and could be a symbol of recovery and return to a normal life [5, 8]. Returning to work may help patients overcome the negative impacts of disease treatments, has a positive financial outcome for patients, and reduces the economic burden of cancer on society [9, 10]. Many cancer survivors are able to return to work [11–13], but are likely to encounter significant difficulties as a result of reduced working hours, fatigue, and exhaustion [3, 14–18]. In a systematic review, Spelten et al. [5] evaluated 14 studies published from 1985 to 1999 and found that 62 % (range, 30–93 %) of cancer survivors had returned to work. Similarly, Mehnert et al. [13] evaluated 64 studies published from 2000 to 2009 and found that 62 % (range, 24–94 %) of cancer survivors had returned to work. Several studies reported that an average of 60 to 67 % of patients who had worked before their cancer diagnosis returned to work after the initial treatments [3–7]. In Japan, Ito et al. [19] reported that 75.8 % of cancer survivors had returned to work. The present study thus aimed to clarify the status of returning to work among gynecologic cancer survivors. A high proportion of patients (71.3 %) in this study returned to work at the same workplace. Furthermore, 83.9 % of gynecologic cancer survivors who had worked before their cancer diagnosis returned to work, included those who experienced a job change after treatment. The findings of this study are consistent with the results obtained for patients with other diseases and confirm that a significant percentage of survivors quit working for cancer-related reasons.

In this study, 16.1 % of patients did not return to work, and 12.6 % patients changed their job. We investigated the employment patterns and status of returning to work in patients with gynecologic cancer. No return to work (including because the business closed) occurred in 9.7 % of self-employed patients, 5.9 % of regularly employed patients, and 30.5 % of non-regularly employed patients. Those who changed their jobs constituted 11.1 % of publicly employed patients, 14.7 % of regularly employed patients, and 15.9 % of non-regularly employed patients. Furthermore, among patients who were able to return to the same workplace, 90.3 % were self-employed, 88.9 % were publicly employed, 79.4 % were regularly employed, and 53.7 % were non-regularly employed.

In patients with breast cancer, returning to work is associated with several risk factors such as sociodemographic factors, disease-related factors, treatment-related factors, psychological factors, and work-related factors [20]. We investigated the correlations between each characteristic and no return to work or job change. Our results showed that non-regular employment was the variable most likely to negatively affect return to work and job change. Interestingly, the findings of this study confirm that a significant percentage of survivors quit working for reasons related to their employment pattern. Among all employment patterns (non-regular, self-, public, and regular employment), significantly more patients experienced job changes than returned to work at the same workplace on the >6 months reinstatement after treatment. Among all employment patterns, significantly fewer patients changed jobs than returned to work at the same workplace on work times and personal income after treatment. The findings of this study confirm that change the job patients were significant high percentage of survivors extended reinstatement or considerably reduced their work hours and income.

We acknowledge that our study has some limitations. The number of patients was relatively small, and the examination was performed at a single facility. Further prospective studies involving more patients and facilities would provide more definitive data with which to clarify the significance of our findings.

## Conclusions

Gynecologic cancer is one of the most common malignant diseases in working-age women. This study has revealed the status of returning to work in patients with gynecologic cancer. The present findings suggest that non-regular employment was the variable most likely to negatively affect return to work and job change in gynecologic cancer survivors. A high proportion of patients (71.3 %) in this study returned to work at the same workplace. However, 53.7 % of non-regularly employed patients continued to be employed at the same workplace. Non-regular employment was the variable most likely to negatively affect return to work and job change. Prevention of not returning to work and changing jobs may be one of the most important factors for positive relationships with the patient, employer, and society. Social support should be established to ensure satisfactory return to work.

## Abbreviations

AUC, area under the curve; ROC, receiver operating characteristic
